# Supplementary material for: Structural and Socio-Spatial Determinants Influencing Care and Survival of Patients with a Pancreatic Adenocarcinoma: Results of the PANDAURA Cohort
Source: Cancers (Basel). 2022 Nov 3;14(21):5413. doi: 10.3390/cancers14215413 (PMC9658615; doi:10.3390/cancers14215413)
Supplement: Supplementary file 1 [file cancers-14-05413-s001.zip › cancers-1978546-supplementary.pdf]

cancers MDPI

Table S1. Overall survival univariate analysis in the global cohort.

Variables		n	HR	IC95	*p*-value
**Clinical characteristics**					
**Sex**	Female	536	0.970	[0.81-1.16]	0.7331
	Male				
**Age**	Median	536	1.024	[1.02-1.03]	**<0.0001**
	≤ 70	536			**0.0001**
	> 70		1.408	[1.18-1.68]	
**BMI**	< 20	536			0.3196
	[20-24]		0.789	[0.59-1.06]	
	[25-29]		0.911	[0.67-1.24]	
	≥ 30		0.827	[0.58-1.17]	
**Smoking status**	No smoking	536			0.0849
	Weaned smoking		0.837	[0.66-1.06]	
	Active smoking		0.782	[0.61-1.01]	
**History of other cancer**	No	536			0.3777
	Yes		1.116	[0.87-1.42]	
**Presence of diabetes**	No	536			0.6624
	Yes		1.044	[0.86-1.26]	
**Circumstances of cancer diagnosis**	Incidental	536	0.786	[0.59-1.05]	0.1069
	Presence of symptoms				
**Stage at the diagnosis**	Resectable	536			**<0.0001**
	Borderline/locally advanced		1.733	[1.36-2.21]	
	Metastatic		5.726	[4.41-7.44]	
**Histological grade**	Well differentiated	305			**0.0170**
	Moderately differentiated		1.318	[1.00-1.74]	
	Poorly differentiated		1.697	[1.17-2.45]	
**Delay between onset symptoms and first CT scan (days)**	≤ 15	478			0.29
	> 15		1.159	[0.97-1.39]	
**Delay between CT scan and first treatment (days)***	All-treated patients	436			**<0.0001**
	≤ 21				
	> 21		0.534	[0.44-0.65]	
	Metastatic subgroup	131			**0.0085**
	≤ 21		2.02	[1.18-3.45]	
	> 21				
	Resectable subgroup	115			0.8470
	≤ 21		0.95	[0.58-1.56]	
	> 21				
**Surgical features**					
**Resection margins status**	R0	146			0.5125
	R1		1.175	[0.73-1.90]	
**Surgery-related adverse events**	No	179			0.6989
	Yes		1.068	[0.77-1.49]	
**Number of pancreatic surgeries per center per year**	< 5	177	2.020	[1.23-3.33]	**0.0180**
	5 - 20		1.306	[0.92-1.85]	
	> 20				
**Number of cancer surgeries (all types included) per center per year**	≤ 100	177	1.150	[0.69-1.91]	0.5871
	> 100				
**Socio-spatial determinants**					
**Localized Potential Accessibility**	≤ 46.7	536	1.005	[0.76-1.33]	0.1368
	[46.8 ; 63.8]		1.371	[1.04-1.81]	
	[63.9 ; 74.3]		1.199	[0.91-1.57]	
	[74.4; 86.4]		1.109	[0.83-1.48]	

	> 86.4				
**French deprivation index**	≤ -1.3	536	1.031	[0.79-1.35]	0.1065
	[-1.2 ; -0.5]		0.728	[0.55-0.96]	
	[-0.4 ; 0.3]				
	[0.4 ; 1]		0.901	[0.68-1.19]	
	> 1		0.877	[0.67-1.15]	
**Population density (people/km²)**	≤ 83.7	536			0.6611
	[83.8 ; 254.7]		0.983	[0.75-1.30]	
	[254.8 ; 798.1]		0.870	[0.66-1.15]	
	[798.2 ; 2159.9]		1.044	[0.79-1.38]	
	> 2159.9		1.057	[0.80-1.39]	
**GeoClasH classification**	Wealthy Metropolitan Areas areas	536			0.6885
	Precarious Population Districts areas		0.871	[0.70-1.08]	
	Residential Outskirts		0.986	[0.74-1.31]	
	Agricultural and Industrial Plains		0.973	[0.58-1.64]	
	Rural Margins		1.008	[0.72-1.40]	
**Spatial accessibility (time travel to an expert center in min)**	≤ 13	536			0.2372
	[14 ; 36]		0.773	[0.59-1.02]	
	[37 ; 58]		0.879	[0.67-1.15]	
	[59 ; 79]		0.906	[0.69-1.18]	
	> 79		0.755	[0.57-0.99]	

P-values < 0.05 are marked in bold characters. * Delay between CT scan and first treatment (days) is only analysed in treated patients excluding exclusive best supportive care (n=438). Abbreviations: BMI= body mass index; n: number of patients with data available for analysis.

Table S2. Impact of clinical features and delay on overall survival in the treated cohort after multivariate analyses. Final multivariate model including 431 patients (exclusion of patients with exclusive best supportive care).

Variables	Value	HR	IC95	*p*-value
**BMI**	<20			NS(1)
	[20-24]			
	[25-29]			
	≥ 30			
**Age (years)**	≤ 70			0.0011
	>70	1.392	[1.14-1.70]	
**Circumstances of cancer diagnosis**	Incidental diagnosis			0.0116
	Presence of symptoms	1.545	[1.10-2.17]	
**Presence of diabetes**	No			NS(3)
	Yes			
**Sex**	Female			NS(2)
	Male			
**Stage at the diagnosis**	Resectable			<0.0001
	Borderline/locally advanced	1.701	[1-31-2.19]	
	Metastatic	5.344	[3.99-7.16]	
**Delay CT-Scan – first treatment**	≤ 21 days			0.4220
	> 21 days	0.884	[0.66-1.19]	

The number presented between parenthesis in the p-value column corresponds to the rank of the variable (rank) in the multivariate analysis based on its *p*-value. *P*-values < 0.05 are marked in bold characters. Abbreviations: BMI= body mass index

Table S3. Surgery-related adverse events and mortality according to volume of pancreatic surgery per center.

	Number of surgeries per center per year								All		Test
	Missing data N=3		5-20 N=69		<5 N=21		>20 N=88		N=181		
Surgery-related adverse events											
Missing data	0		1		0		0		1		Chi-2 $P = 0.927$
No	2	(66.7%)	43	(63.2%)	14	(66.7%)	58	(65.9%)	117	(65.0%)	
Yes	1	(33.3%)	25	(36.8%)	7	(33.3%)	30	(34.1%)	63	(35.0%)	
Duration of hospitalization (days)											
N	3		69		21		88		181		Kruskal-Wallis $P = 0.657$
Mean (SD)	19.67 (7.51)		18.29 (15.66)		16.10 (12.08)		16.32 (8.76)		17.10 (12.15)		
Median (min; max)	20.00 (12.00; 27.00)		14.00 (3.00; 116.00)		13.00 (3.00; 58.00)		15.00 (2.00; 58.00)		14.00 (2.00; 116.00)		
IQR [Q1 - Q3]	[12.00 - 27.00]		[10.00 - 20.00]		[10.00 - 17.00]		[10.00 - 19.00]		[10.00 - 20.00]		
Transfer in intensive care unit											
No	2	(66.7%)	57	(82.6%)	16	(76.2%)	77	(87.5%)	152	(84.0%)	Chi-2 $P = 0.393$
Yes	1	(33.3%)	12	(17.4%)	5	(23.8%)	11	(12.5%)	29	(16.0%)	
Death within 30 days after surgery	0	(0.0%)	3	(4.3%)	2	(9.5%)	1	(1.1%)	6	(3.3%)	Fisher test $P = 0.11$
